# Targeted Sequencing Revealed Distinct Mutational Profiles of Ocular and Extraocular Sebaceous Carcinomas

**DOI:** 10.3390/cancers13194810

**Published:** 2021-09-26

**Authors:** Hee Young Na, Jeong Hwan Park, Sun Ah Shin, Sejoon Lee, Heonyi Lee, Heejoon Chae, HoKyung Choung, Namju Kim, Jin-Haeng Chung, Ji Eun Kim

**Affiliations:** 1 Department of Pathology, Seoul National University College of Medicine, Seoul 03080, Korea; 66040@snubh.org (H.Y.N.); ruvit21@snu.ac.kr (J.H.P.); 13623@ncc.re.kr (S.A.S.); 2 Department of Pathology and Translational Medicine, Seoul National University Bundang Hospital, Seongnam 13620, Korea; 3 Department of Pathology, Seoul Metropolitan Government-Seoul National University Boramae Medical Center, Seoul 07067, Korea; 4 Department of Pathology, National Cancer Center, Goyang 10408, Korea; 5 Precision Medicine Center, Seoul National University Bundang Hospital, Seongnam 13620, Korea; sejoonlee@snubh.org; 6 Bioinformatics Collaboration Unit, Department of Biomedical Systems Informatics, Yonsei University College of Medicine, Seoul 03722, Korea; hylee4161@yuhs.ac; 7 Division of Computer Science, Sookmyung Women’s University, Seoul 04312, Korea; heechae@sookmyung.ac.kr; 8 Department of Ophthalmology, Seoul Metropolitan Government-Seoul National University Boramae Medical Center, Seoul 07067, Korea; hokyung2@snu.ac.kr; 9 Department of Ophthalmology, Seoul National University Bundang Hospital, Seongnam 13620, Korea; resourceful@hanmail.net

**Keywords:** sebaceous carcinoma, genetics, next-generation sequencing

## Abstract

**Simple Summary:**

The molecular pathogenesis of sebaceous carcinoma is largely unknown so far. The main aim of our study was to investigate genetic alterations in ocular and extraocular sebaceous carcinoma in Korean patients. By applying targeted next-generation sequencing, we demonstrated a distinct mutational profile in ocular and extraocular sebaceous carcinoma. In addition, clinically actionable variants involving tyrosine kinase genes and homologous recombination deficiency-associated genes were detected, suggesting the possibility of targeted therapeutic trials in intractable sebaceous carcinoma cases.

**Abstract:**

The biological behavior of sebaceous carcinoma (SeC) is relatively indolent; however, local invasion or distant metastasis is sometimes reported. Nevertheless, a lack of understanding of the genetic background of SeC makes it difficult to apply effective systemic therapy. This study was designed to investigate major genetic alterations in SeCs in Korean patients. A total of 29 samples, including 20 ocular SeCs (SeC-Os) and 9 extraocular SeCs (SeC-EOs), were examined. Targeted next-generation sequencing tests including 171 cancer-related genes were performed. *TP53* and *PIK3CA* genes were frequently mutated in both SeC-Os and SeC-EOs with slight predominance in SeC-Os, whereas the *NOTCH1* gene was more commonly mutated in SeC-EOs. In clinical correlation, mutations in *RUNX1* and *ATM* were associated with development of distant metastases, and alterations in *MSH6* and *BRCA1* were associated with inferior progression-free survival (all *p* < 0.05). In conclusion, our study revealed distinct genetic alterations between SeC-Os and SeC-EOs and some important prognostic molecular markers. Mutations in potentially actionable genes, including *EGFR*, *ERBB2*, and mismatch repair genes, were noted, suggesting consideration of a clinical trial in intractable cases.

## 1. Introduction

Sebaceous carcinoma (SeC) is a rare but aggressive neoplasm that is relatively prevalent in the Asian population [1,2,3]. The cellular origins of SeC are secretory cells producing oily substance or ductal cells of the sebaceous gland [4]. Diagnosis of SeC is very challenging because of the anatomical proximity and histological similarity to hair follicle or sweat glands. It predominantly occurs in periocular areas, where Meibomian glands are enriched, but extraocular sites, including the head and neck region, and less frequently the trunk or breast, can also be involved [1]. Wide excision is the mainstay of treatment for both ocular sebaceous carcinomas (SeC-Os) and extraocular sebaceous carcinomas (SeC-EOs) [5]. However, locally aggressive SeC-O often requires extensive surgery, including orbital exenteration, leading to high morbidity [6,7]. Radiotherapy can be considered in unresectable, locally recurrent, or residual disease [5]. The cancer-specific mortality rate of SeC is 6–22% [8,9,10], and disease progression, including locoregional recurrence and distant metastasis, is reported in 4–28% [1,11,12]. Systemic therapy, including conventional chemotherapy, immunotherapy, and targeted therapies, can be considered in locally aggressive or metastatic SeCs, although the outcome data are scarce [5,13]. The use of conventional chemotherapy composed of Adriamycin and cyclophosphamide or platinum-based chemotherapeutic agents with either paclitaxel or 5-fluorouracil and folinic acid has been reported by some researchers [14,15,16]. Immunotherapy for microsatellite unstable SeCs or targeted therapies, such as retinoic acid receptor-β agonist [17], androgen receptor (AR) antagonist [18], and epidermal growth factor receptor inhibitor [19], are other potential options. However, no single effective targeting agent has been established, primarily due to the lack of understanding of the molecular pathogenesis of this tumor.

Next-generation sequencing (NGS) has opened a new era of high-throughput biology and has enabled researchers to identify large-scale genomic alterations in a relatively short time, contributing to the fine-tuning of cancer management. Accordingly, several outstanding studies of a comprehensive molecular analysis of SeC using NGS were recently published [20,21,22]. Some researchers have proposed that SeC shows genetic signatures similar to those of cutaneous squamous cell carcinoma, demonstrating alterations of *TP53*, *NOTCH1* and *NOTCH2* [20,21,22]. However, our previous reports revealed that the molecular pathogenesis of SeC could be compared with that of breast carcinoma because of its ontogenetic proximity and hormone-responsive growth, reflecting the similarity between sebaceous glands and mammary glands [23,24]. Moreover, most molecular studies were performed in Western cohorts, and molecular pathologic data of SeC in Asian populations require further investigation. In the present study, we investigated the genetic profile of SeCs in Korean patients, focusing on a comparison of cutaneous squamous cell carcinoma and mammary carcinoma, expecting to find a new therapeutic target.

## 2. Materials and Methods

### 2.1. Patients and Specimens

Surgically resected formalin-fixed paraffin-embedded (FFPE) tissue specimens of 29 SeC cases were obtained from 26 selected patients who were treated at Seoul Metropolitan Government Seoul National University (SMG-SNU) Boramae Medical Center, Seoul National University Bundang Hospital, and Seoul National University from 2000 to 2015. Among these 29 tumors, there were 26 primary lesions, 1 locally recurrent SeC-O, 1 locally recurrent SeC-EO, and 1 metastatic SeC-O. Among the 3 locally recurrent or metastatic lesions, locally recurrent SeC-EO and metastatic SeC-O had matched primary lesions. In addition, 2 primary lesions (1 SeC-O and 1 SeC-EO) from 1 suspected Muir–Torre syndrome (MTS) patient were included. Clinicopathologic information, including age, sex, and stage, was retrieved from the electronic medical records and pathology reports. This study was approved by the Institutional Review Board (IRB) at SMG-SNU Boramae Medical Center (IRB No. 16-2017-17-031).

### 2.2. DNA Extraction and NGS

Targeted DNA sequencing was performed using the Axen Cancer Panel 2 (Macrogen, Korea) composed of 171 cancer-associated genes (Table S1). After reviewing each slide, tumor areas were carefully chosen for manual macrodissection. The tumor purity ranged from 40% to 60%. To extract the genomic DNA, a QIAamp^®^ DNA FFPE Tissue kit (Qiagen, Hilden, Germany) was used. A total of 200 ng of DNA was used for library generation. The hybrid capture method was used for DNA library preparation and target enrichment, according to Illumina’s standard protocol using an Agilent SureSelect^XT^ Target Enrichment Kit (Agilent Technologies, Santa Clara, CA, USA). The target region was sequenced using the HiSeq 2500 system (Illumina, San Diego, CA, USA). The average mean depth was ×1100.

### 2.3. Alignment of Reads and Variant Calling

The cutadapt [25] was used to remove adapter sequences from the raw sequencing reads, followed by aligning the reads to the reference genome (GRCh37/hg19) using Burrows–Wheeler Aligner-MEM (BWA-MEM) [26]. Poorly mapped reads with a mapping quality (MAPQ) below 20 were removed using Samtools version 1.3.1 [27]. The MuTect2 algorithm [28] was used to detect somatic mutations, including single nucleotide variants (SNVs) and small insertions and deletions (INDELs). Finally, SnpEff and SnpSift v4.3i [29] with dbNSFP v2.9.3 [30] were used to annotate all of the detected variants.

### 2.4. Single Nucleotide Variant Detection

Since the present study did not include the matched germline samples, variants satisfying the following criteria were excluded to reduce the proportion of false-positive or possible germline variants: (i) variants with less than 5% allele frequency and <100× read depth; (ii) variants with an allele frequency >1% in the Exome Aggregation Consortium (ExAC) East Asian database [31]; (iii) all synonymous, intronic, 3′- and 5′-untranslated region (UTR) variants; and (iv) variants previously reported to be benign or likely benign with ascertained criteria in the ClinVar archive [32]. Finally, pathogenic mutations described in the COSMIC [33] database and truncation mutations of tumor suppressor gene were selected in analysis.

### 2.5. Immunohistochemistry (IHC) for Molecular Subtypes

A total of 22 out of 29 SeCs were classified into 5 molecular subtypes in a similar way to breast cancer, as described in our previous study (luminal 1, luminal 2, HER2, all-negative, and core basal) (Table S2) [24]. The remaining 7 cases had no available tissue for IHC study. An automated immunostainer (Ventana BenchMark XT, Tucson, AZ, USA) was used to perform the IHC for anti-HER2 (Dako, Carpinteria, CA, USA), antiandrogen receptor (anti-AR; Thermo Scientific, Carlsbad, CA, USA), anti-ER (Dako), anti-PR (Dako), anti-CK5/6 (Dako), and anti-Ki67 (Dako) antibodies. The interpretation of HER2 IHC results was based on the 2013 American Society of Clinical Oncology (ASCO)/College of American Pathologists (CAP) guidelines [34]. Finally, scores of 2+ and 3+ were considered positive for HER2. For hormone receptors (HRs), including AR, ER, and PR, it was considered positive when ≥1% of the tumor cell nuclei were stained. CK5/6 was scored when ≥50% of the tumor cells were strongly immunoreactive [24].

### 2.6. Bioinformatics Analysis

We used the maftools [35] package and R version 3.6.3 (http://www.r-progect.org/, accessed on 10 September 2021) to generate an oncoplot, which depicts the top 20 mutated genes in SeCs. We expanded the altered genes of each SeC by weighting frequent recurrent mutations on String with adjustment of settings (associated gene; except textmining, high confidence (0.700), no more than 5 interactors). Then, we evaluated protein–protein interaction (PPI) and conducted gene ontology (GO) pathway analysis using String v. 11.0 [36].

### 2.7. Statistical Analysis

Clinicopathologic variables were correlated with the presence or the frequency of genomic alterations in SeC-Os and SeC-EOs. The differences were analyzed by using Pearson’s chi-square test, Fisher’s exact test, or the Mann–Whitney U test. Progression-free survival (PFS) of SeC patients was analyzed by Kaplan–Meier survival curves. All the statistical analyses were performed using the SPSS (v22.0) software package (SPSS, Chicago, IL, USA). All *p*-values were two-sided, and a *p*-value of less than 0.05 was considered statistically significant.

## 3. Results

### 3.1. Patient Characteristics

The clinical profile of all patients is summarized in the Table 1. Among 26 patients, there were 18 patients with SeC-O and 8 patients with SeC-EO. The median age of the patients with SeC-O and SeC-EO was 64 years (range, 41–84 years) and 55 years (range, 42–70 years), respectively. A total of 15 (53.6%) and 4 (14.3%) SeC-Os were categorized as pathological T (pT) categories 1–2 and 3–4, respectively, while all 9 (100.0%) SeC-EOs were staged as pT1–2. Disease progression was noted in 8 (30.8%) patients with SeC-Os and 1 (12.5%) patient with SeC-EOs. None of the patients died during the follow-up period (median, 29 months; range, 1–132 months).

### 3.2. Genetic Alterations in SeC-Os and SeC-EOs

**Figure 1:**
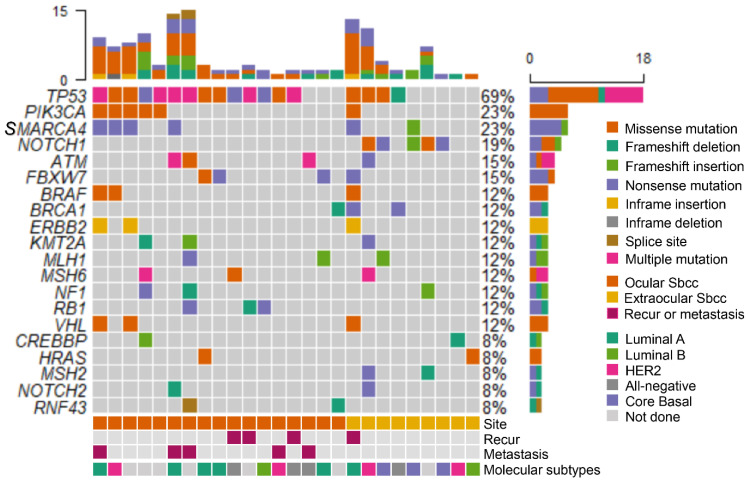
Oncoplot of ocular and extraocular sebaceous carcinomas. *TP53* and *PIK3CA* genes were frequently mutated in ocular sebaceous carcinomas, while the *NOTCH1* gene was more commonly mutated in extraocular sebaceous carcinomas.

Targeted sequencing in 29 SeCs revealed 129 pathogenic mutations in 34 different genes (Table 2 and Figure 1). On average, 4.44 pathogenic mutations per tumor were observed (range, 0–15). The number of pathogenic mutations did not differ between the SeC-Os and SeC-EOs (*p* = 0.864).

In SeC-Os, the highest number of pathogenic mutations was detected in *TP53*, which harbored a total of 29 mutations in 13 cases (65.0%). Among 29 mutations, 21 were missense mutations, and 7 were nonsense mutations. The remaining 1 alteration occurred in the spliced acceptor site (c.920-1G > A). A total of 6 mutations in *PIK3CA* were observed in 4 cases (20.0%). *SMARCA4*, *ATM*, *FBXW7*, and *RB1* were mutated in 3 cases (15.0%). In SeC-EOs, the most commonly mutated gene was *NOTCH1*, of which a total of 6 alterations were detected in 5 tumors (55.5%). There were 6 missense mutations, 3 nonsense mutations, and 1 frameshift mutation. In 3 (33.3%) tumors, a total of 8 *TP53* alterations were observed, of which 7 were missense mutations, and the remaining 1 was a frameshift mutation. For *BRCA1*, *MSH2*, and *SMARCA4*, a total of 2 cases (22.2%) showed pathogenic variants, respectively (Table 2). Mutations in other potentially targetable genes, such as *BRAF*, *ERBB2*, and *EGFR*, were also detected (Table 2). Mutations in BRAF were observed in 2 (10.0%) SeC-O and 1 (11.1%) SeC-EO cases, all of which were p.N581S. All 3 cases harbored mutations in either ERBB2 (p.A775_G776insYVMA) or EGFR (p.L747_E749del). In addition, HRAS alterations were found in 1 (5.0%) SeC-O and 1 (11.1%) SeC-EO, and KMT2A alteration was observed in 2 (10.0%) SeC-O and 1 (11.1%) SeC-EO cases.

Compared with SeC-Os, mutations in *NOTCH1* were significantly more enriched in SeC-EOs (*p* = 0.001). Although *TP53* was more frequently mutated in SeC-Os than in SeC-EOs, and *RB1* was mutated only in SeC-O cases, the difference was not statistically significant (*p* = 0.532). Likewise, the mutations in *MSH2* were only observed in SeC-EOs, but the difference was not significant (*p* = 0.234).

**Figure 2:**
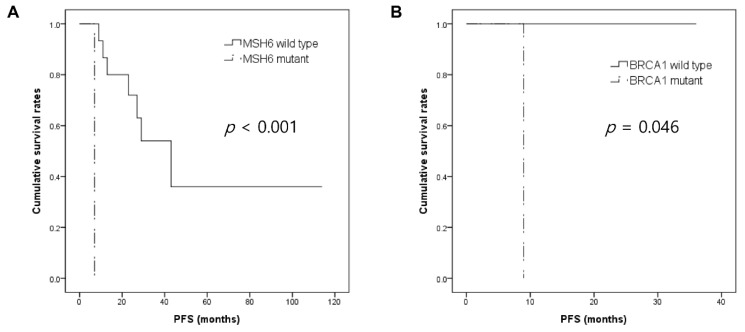
Kaplan–Meier analysis revealed (**A**) *MSH6* mutation, and (**B**) *BRCA1* mutation was associated with inferior progression-free survival in ocular and extraocular sebaceous carcinomas, respectively.

Mutations in *RUNX1* were observed in 2 SeC-Os, and mutations in *ATM* were identified in 3 SeC-Os and 1 SeC-EO. Although the frequency of mutations in these 2 genes were low, they were associated with the development of distant metastases during the follow-up period (*p* = 0.046, *p* = 0.028). However, cases harboring mutations in RUNX1 and ATM were not associated with shorter PFS in Kaplan–Meier analysis (both *p* > 0.05) (Figure S1). In SeC-Os, cases harboring *MSH6* alterations showed significantly shorter PFS (*p* < 0.001), whereas in SeC-EOs, cases with *BRCA1* mutations presented inferior PFS (*p* = 0.046) (Figure 2).

### 3.3. PPI in SeCs

**Figure 3:**
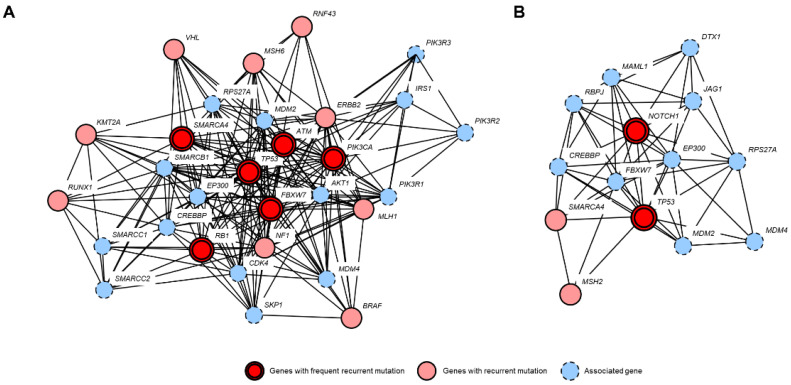
Analysis of PPI based on genes with recurrent mutation and associated genes. (**A**) Ocular sebaceous carcinoma (SeC-O). (**B**) Extraocular sebaceous carcinoma (SeC-EO). The TP3 pathway, SWItch/Sucrose Non-Fermentable (SWI/SNF) pathway, and MMR pathway were shared by both SeC-O and SeC-EO. The PI3K/AKT/mTOR pathway (*PIK3CA*) and Notch signaling pathway (*NOTCH1*) were unique to SeC-O and Sec-EO, respectively.

Analysis of PPI based on genes with recurrent mutations and associated genes suggested that the TP3 pathway, SWItch/Sucrose Non-Fermentable (SWI/SNF) pathway, and MMR pathway were mainly responsible for the pathogenesis of both SeC-O and SeC-EO (Figure 3). Additionally, the PI3K/AKT/mTOR pathway (*PIK3CA*) and Notch signaling pathway (*NOTCH1*) were considered other tumorigenic pathways in SeC-O and Sec-EO, respectively (Figure 3). In GO analysis, mutated and associated genes were enriched in androgen receptor binding or signaling pathway, and p53 binding pathway in both SeC-O and SeC-EO (Table S3 and Figure S2B and Figure S3A,B). In SeC-O, genes associated with ERBB2 signaling pathway were enriched (Table S3 and Figure S2A, whereas genes associated with cellular response to ultraviolet (UV) were enriched in SeC-EO, suggesting a possible role of UV in SeC-EO tumorigenesis (Table S3 and Figure S3A).

### 3.4. Genomic Alterations according to the Molecular Subtypes

The molecular subtypes were determined in a total of 22 cases with available tissue for IHC and FISH analysis. In SeC-Os, 6 (42.9%), 2 (14.3%), 2 (14.3%), 4(28.6%), and 0 (0.0%) cases were classified as luminal 1, luminal 2, HER2, all-negative, and core basal type (Table 1). In SeC-EOs, 1 (14.3%), 1 (14.3%), 2 (28.6%), 1 (14.3%), and 3 (24.9%) cases were categorized as luminal 1, luminal 2, HER2, all-negative, and core basal type (Table 1). The genes that harbored pathogenic mutations in more than 2 cases are summarized according to the molecular subtypes in Table 3. *TP53* was the most frequently mutated gene across the 5 subtypes. *FBXW7* mutations were significantly enriched in the luminal 1 subtype compared with the other subtypes (*p* = 0.006). Likewise, mutations in *NOTCH1* were observed only in the core basal subtype (*p* = 0.003). Although mutations in *TP53* and *SMARCA4* were more common in the luminal 1 subtype, the differences were not significant (*p* > 0.05). Mutations involving ERBB2 were detected only in the luminal 1 subtype.

### 3.5. Alterations in MMR Genes and Lynch Syndrome

One patient clinically diagnosed with Muir–Torre syndrome (MTS) was included in this study. This patient had a microsatellite instability (MSI)-high colon cancer, followed by early gastric carcinoma and SeC-O 2 years later, and then was diagnosed with metachronous SeC-EOs. The NGS study performed in 2 SeCs revealed a common mutation of *MLH1* (p.M587Hfs*6) with a variant allele frequency (VAF) of 45.5% in SeC-O and 60.16% in SeC-EO (Table 4). Considering the VAF of the *MLH1* mutation, this mutation was highly suggestive of a germline variant. Interestingly, his SeC-O and SeC-EO had no overlapping pathogenic variants other than the *MLH1* mutation (Table 4).

In addition to the patient described above, 5 other patients had alterations in MMR genes (Table S4). The number of patients with mutations in *MLH1*, *MSH2*, and *MSH6* was 1, 1, and 2, respectively. Mutations in both *MSH2* and *MSH6* were detected in the remaining 1 patient. Only 1 case revealed a possible germline variant in *MSH6* (p.F1088Lfs*5) with a VAF of 47.66% but no other accompanying internal malignancies conforming to MTS. Except for that case, the VAFs of the other mutations were not suggestive of germline mutations. Alterations in MMR genes were associated with mutations involving KMT2A (*p* = 0.010) and NF1 (*p* = 0.052) with borderline significance. The number of pathogenic mutations was higher in tumors with MMR gene alterations than in others with borderline significance (*p* = 0.053).

### 3.6. Comparison among Primary and Locally Recurrent or Metastatic Lesions

**Figure 4:**
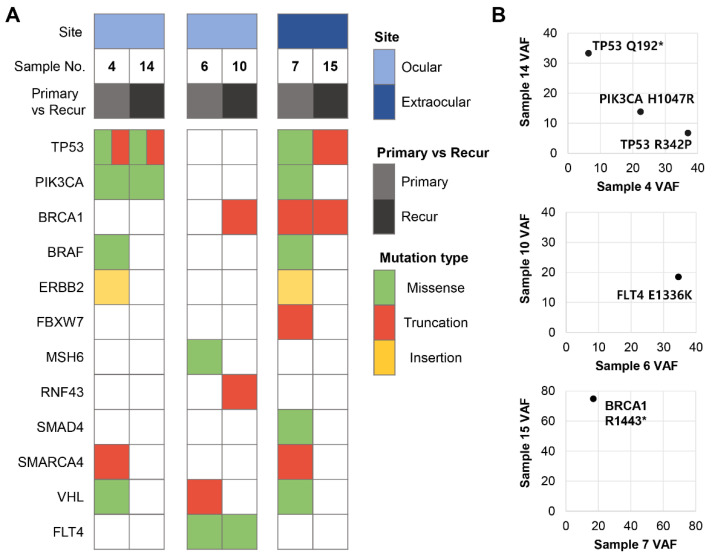
Comparison between primary and recurrent lesions. (**A**) Two patients showed common pathogenic mutations in *TP53*, *PIK3CA*, and *BRCA1*. Sample 6 and 10 from other patient harbored a common *FLT4* mutation, which is of unknown clinical significance. (**B**) The frequency of mutated *TP53* and *BRCA1* alleles increased in recurrent lesion in sample 14 and 15.

There were 3 cases in which the mutation profile of the primary site and recurrent or metastatic tumors could be compared (Figure 4). Among them, 2 cases revealed common pathogenic mutations between the primary sites and others. Samples 4 (the original site) and 14 (the metastatic lesion) shared mutations involving *PIK3CA* and *TP53*. Likewise, in sample 7 and its recurrent lesion (sample 15), the same *BRCA1* nonsense mutation was detected (Figure 4). Notably, the number of pathogenic mutations decreased in the recurrent lesions (samples 14 and 15) compared with the primary lesions (samples 4 and 7). However, the tumor population harboring *TP53* p.Q192* and *BRCA1* p.R1443* mutations increased in the recurrent lesions (Figure 4). On the other hand, in 1 patient out of 3, there was no common pathogenic mutation shared by both primary (sample 6) and recurrent tumors (sample 10), with the exception of the mutation in *FLT4* (p.E1336K), which is a variant of undetermined significance.

## 4. Discussion

In the current study, we comparatively analyzed the mutational profile of SeC-Os and Sec-EOs using targeted NGS. Our results suggest that SeC-Os and SeC-EOs are related but distinct entities with different molecular pathogeneses. SeC showed frequent alterations involving *TP53*, *NOTCH1*, *PIK3CA*, and *SMARCA4*, whereas SeC-Os revealed mutations in *TP53*, *PIK3CA*, *FBXW7*, and *RB1*. Notably, *NOTCH1* mutations were exclusively found in SeC-EOs. Given that most of these *NOTCH1* mutations are related to a loss of function, *NOTCH1* may play a role as tumor suppressor in SeC-EOs. On the other hand, *FBXW7*, which is known to negatively regulate *NOTCH* [37] and leads to activation of the Notch signal when mutated, was more commonly altered in SeC-Os. Therefore, direct or indirect activation of the Notch signaling pathway appears to be involved in the tumorigenesis of both SeC-Os and SeC-EOs. However, genes that are related to the PI3K/AKT/mTOR pathway, such as *PIK3CA* [38], *FBXW7* [39], *PTEN*, and *TSC1* [40], were more frequently mutated in SeC-Os. PPI analysis suggested that the TP53 pathway, SWI/SNF pathway, and MMR pathway were the main pathogenic pathways in SeCs.

Our results are in line with the previous studies, which have also demonstrated frequent activation mutations in the PI3K/AKT/mTOR pathway in SeCs [20,21,22]. The PI3K/AKT/mTOR pathway plays an important role in controlling cell growth and proliferation [41]. Activation of this pathway contributes to cell proliferation by stimulating downstream targets [41]. This phenomenon is also well known in other tumors, including breast [42], ovarian [43], and urothelial cancers [44]. North et al. also suggested a distinct pathogenesis for SeC-Os and SeC-EOs; mutations in *TP53*, *NOTCH1*, *NOTCH2*, *ZNF750*, and *RREB1* are specific to SeCs, whereas truncation mutations in *NOTCH1* are concentrated in SeC-EOs [20]. Notably, SeC-Os were enriched with mutations in *ZNF750*, while SeC-EOs showed a high mutation burden associated with MSI and UV damage [20]. Since *ZNF750* is known as a transcription factor involved in keratinocyte differentiation [45,46], their findings provide support for the theory that the pathogenesis of SeC is closely related to epidermal carcinoma. However, *ZNF750* was not included in our gene panel, and there are other studies that have published some controversial results [22]. Except for *ZNF750*, the mutational profile of SeCs in our cohort was similar to that of cutaneous squamous cell carcinoma (SqCC), in which *TP53* and *NOCTH1/2* are characteristically mutated [47,48]. Basal cell carcinoma (BCC) is a frequent mimicker of SeC because of its similar morphologic characteristics and cellular origin (folliculosebaceous germinative cells). However, BCC is known to show a different mutation pattern from SeC; sonic hedgehog pathway genes, such as *PTCH1*, *SMO*, or *SUFU*, were the mainly altered genes in BCC in addition to *TP53*, unlike SeCs [49]. These results suggest that the molecular characteristics of SeCs are more akin to cutaneous SqCCs than to BCCs.

Our group previously proposed five molecular subtypes of SeCs according to the HRs, HER2, and CK5/6, in a similar way to breast cancer: luminal 1, luminal 2, HER2, all-negative, and core basal subtypes [24]. While SeC-EOs were more frequently classified as core basal subtypes, SeC-Os prevailed among the other four subtypes. When comparing mutational profiles, *FBXW7* mutations were significantly enriched in the luminal 1 subtype, and mutations in *NOTCH1* were observed only in the core basal subtype (*p* = 0.003). Although SeCs and breast cancer are similar in that *PIK3CA* and *TP53* are the most common mutations, there was little overlap between the two tumors in terms of the types of alterations according to each molecular subtype [50,51]. For example, in general, basal-like breast carcinoma has been known to harbor mutations involving *BRCA1*, *CCNE1*, *AKT3*, or *MYC* [50,51]; however, these genes were largely wild type, and *NOTCH1* mutations were relatively common in our SeC-EOs. Wang et al. demonstrated that approximately 13% of triple-negative breast cancer cases carry *NOTCH1* mutations, which are centered in the PEST domain, and ultimately lead to the activation of the Notch signaling pathway [52]. In contrast, none of the six mutations detected in SeC-EOs occurred in the PEST domain, and most of the *NOTCH1* mutations were predicted to damage protein function. Although SeCs can be classified into five immunohistochemical subtypes, their mutational profile did not completely match with these subtypes adopted from breast cancer.

Mutations in MMR genes were found in a total of six patients, including one with classical MTS harboring pathogenic *MLH1* mutations in both SeC-O and SeC-EO. Interestingly, SeC-EO did not share other pathogenic mutations with SeC-O, suggesting multiple individual primary tumors rather than metastasis. In the other five patients with MMR defects, *MLH1*, *MSH2*, and *MSH6* mutations were observed mostly in low VAF, suggesting a somatic origin. Since various types of mutations were found in these cases in addition to MMR defects, it is difficult to determine which of them is the driving mutation. Although we could not confirm the MSI status of these tumors due to tissue unavailability, it is possible that DNA instability induced by MMR defects resulted in numerous mutations. Therefore, we suggest that SeC should also be screened for MSI as for colorectal carcinoma and endometrial carcinoma. Previous studies have also demonstrated that somatic alterations in MMR genes occur especially in SeC-EOs, which show an MSI-high phenotype [20,21].

We had three pairs of primary and matched recurrent or metastatic lesions. In two patients, some of the pathogenic variants of the primary lesions remained in the recurrent lesions. The VAFs of certain variants in the primary lesions were significantly increased (*TP53* p.Q192* in sample 4 and *BRCA1* p.R1443* in sample 7) or decreased (*TP53* p.R342P in sample 4) in recurrent lesions, suggesting an expansion of a specific tumoral subclone that conveys a survival advantage. Notably, samples 6 and 10 did not share any pathogenic variants. Instead, a variant of undetermined significance (*FLT4* p.E1336K) was detected in both lesions. *FLT4*, also known as *VEGFR3*, is an oncogene that mediates cell proliferation, survival, and invasion [53]. Although somatic variants of *FLT4* in solid malignancies are not commonly reported, mutations in colorectal cancer [54] and non-small cell lung cancer [55] have been documented. *FLT4* p.E1336K has not previously been reported; however, the possibility of an oncogenic role can be considered under the above circumstances.

This study identified some potentially targetable alterations in SeCs. First, *PIK3CA* mutations account for 20% (4/20) of SeC-Os, and it can be targeted by PI3K inhibitors similar to hormone receptor-positive breast cancer [56]. Notably, alterations in homologous recombination deficiency (HRD)-related genes, such as *BRCA1*, *BRCA2*, *ATM*, and *KMT2A*, were detected in both SeC-Os and SeC-EOs, suggesting a possible role of poly (ADP-ribose) polymerase inhibitors [57,58]. Second, three patients showed exon 20 insertion mutations in *ERBB2* (p.A775_G776insYVMA), for which a response to pan-HER kinase inhibitors has been reported in various types of cancer [59]. In addition, activating mutations in other kinases, such as *EGFR* (exon 19 deletion) and *BRAF* (p.N581S), were detected, which can also provide grounds for potential targeted therapy [60,61]. Finally, alterations in MMR genes can be an important target for immunotherapy, although further validation of MSI status is required in these tumors [62,63]. Although *NOTCH1* mutations were prominent in SeC-EOs, most alterations were associated with a loss of protein function; therefore, *NOTCH1* inhibitors do not seem promising based on our findings.

One of the novel findings of the present study was that mutations in *RUNX1* (2/29, 6.9%) and *ATM* (4/29, 13.8%) were associated with the development of distant metastasis during the follow-up period. However, cases with mutations in *RUNX1* or *ATM* were not associated with shorter PFS. This might be due to the fact that local recurrences also contribute to disease progression, especially in SeC-Os. *RUNX1* is frequently altered in hematopoietic malignancies [64,65]. Albeit with a lower frequency, mutations in *RUNX1* are also found in solid malignancies, such as breast cancer [66,67,68,69]. Notably, researchers demonstrated that *RUNX1* knockdown induces the activation of transforming growth factor β and WNT signaling pathways, thereby promoting the epithelial–mesenchymal transition (EMT) process [70,71]. Mutations in *ATM* have been reported in various solid tumors, including endometrial cancer, bladder cancer, and colorectal cancer [66,67]. Loss of *ATM* protein expression was associated with poor overall survival in breast cancer [72], cervical cancer [73], and colorectal cancer [74], although the prognostic implications of *ATM* mutation are generally unknown to date. *ATM* signaling is known to induce tumor progression in a nuclear factor kappa B-dependent manner that promotes EMT [75]. Moreover, ATM signaling upregulated the alpha_v_beta_3_ integrin pathway, which can trigger invasive and metastatic potential [76]. As the EMT process is critical in the invasion and development of distant metastasis [77], mutations in *RUNX1* and *ATM* might contribute to developing metastasis. Our findings suggest that mutations in *RUNX1* and *ATM* can serve as prognostic markers in SeCs. In addition, *MSH6* and *BRCA1* mutations were associated with inferior PFS in SeC-Os and SeC-EOs, respectively. For MMR gene defects and BRCA mutations, contradictory results have been reported [78,79,80,81]. Further validation is required to determine the prognostic implications of *MSH6* and *BRCA1* mutations in SeC.

## 5. Conclusions

This is the first study performing NGS in Korean SeC-O and SeC-EO patients. Sequencing revealed a distinct pattern of genetic alterations in SeC-Os and SeC-EOs. Additionally, various potentially targetable alterations were detected, including *PIK3CA*, HRD-related genes, and MMR genes, which could lead to clinical benefit in patients with advanced SeCs.

## Figures and Tables

**Table 1:** Clinicopathological parameters of ocular and extraocular sebaceous carcinomas.

Parameters	Ocular SeC	Extraocular SeC	*p*-Value
Age (mean, range, years) (*n* = 26)	64 (41–84)	55 (42–70)	0.663
Gender (*n* = 26)			0.197
Male	4 (22.2%)	4 (50.0%)	
Female	14 (77.8%)	4 (50.0%)	
Lymphatic invasion (*n* = 29)	3 (15.0%)	1 (11.1%)	>0.999
Neural invasion (*n* = 29)	3 (15.0%)	0 (0.0%)	0.532
pT (*n* = 28)			0.273
pT1 and pT2	15 (53.6%)	9 (100.0%)	
pT3 and pT4	4 (14.3%)	0 (0.0%)	
pN (*n* = 28)			>0.999
pN0	17 (60.7%)	9 (100.0%)	
pN1	2 (7.1%)	0 (0.0%)	
pM (*n* = 28)			>0.999
pM0	18 (64.3%)	9 (100.0%)	
pM1	1 (3.6%)	0 (0.0%)	
Molecular classification (*n* = 22)			0.067
Luminal 1	6 (42.9%)	1 (14.3%)	
Luminal 2	2 (14.3%)	1 (14.3%)	
HER2	2 (14.3%)	2 (28.6%)	
All-negative	4 (28.6%)	1 (14.3%)	
Core basal	0 (0.0%)	3 (42.9%)	
Local recur (*n* = 26)	4 (15.4%)	1 (12.5%)	>0.999
Distant metastasis during F/U (*n* = 26)	6 (23.1%)	0 (0.0%)	0.132
Disease progression (*n* = 26)	8 (30.8%)	1 (12.5%)	0.190
Progression-free survival (months)	24 (2–114)	24 (1–36)	0.322
F/U duration (months)	31 (2–132)	26 (1–69)	0.083

SeC, sebaceous carcinoma; F/U, follow-up.

**Table 2:** List of genes that harbor mutations in ocular and extraocular sebaceous carcinomas.

Gene	Ocular SeC (*n* = 20)	Gene	Extraocular SeC (*n* = 9)
*TP53*	13 (65.0%)	*NOTCH1*	5 (55.5%)
*PIK3CA*	4 (20.0%)	*TP53*	3 (33.3%)
*SMARCA4*	3 (15.0%)	*MSH2*	2 (22.2%)
*ATM*	3 (15.0%)	*SMARCA4*	2 (22.2%)
*FBXW7*	3 (15.0%)	*ATM*	1 (11.1%)
*RB1*	3 (15.0%)	*BAP1*	1 (11.1%)
*BRAF*	2 (10.0%)	*BRAF*	1 (11.1%)
*ERBB2*	2 (10.0%)	*BRCA1*	1 (11.1%)
*MLH1*	2 (10.0%)	*CREBBP*	1 (11.1%)
*RUNX1*	2 (10.0%)	*DNMT3A*	1 (11.1%)
*VHL*	2 (10.0%)	*ERBB2*	1 (11.1%)
*MSH6*	2 (10.0%)	*FBXW7*	1 (11.1%)
*RNF43*	2 (10.0%)	*FGFR2*	1 (11.1%)
*KMT2A*	2 (10.0%)	*HRAS*	1 (11.1%)
*NF1*	2 (10.0%)	*KMT2A*	1 (11.1%)
*APC*	1 (5.0%)	*MLH1*	1 (11.1%)
*BRCA1*	1 (5.0%)	*MSH6*	1 (11.1%)
*BRCA2*	1 (5.0%)	*NF1*	1 (11.1%)
*CREBBP*	1 (5.0%)	*NOTCH2*	1 (11.1%)
*EGFR*	1 (5.0%)	*PIK3CA*	1 (11.1%)
*GNAS*	1 (5.0%)	*PIK3R1*	1 (11.1%)
*HRAS*	1 (5.0%)	*SMAD4*	1 (11.1%)
*IDH1*	1 (5.0%)	*VHL*	1 (11.1%)
*MSH2*	1 (5.0%)		
*NOTCH2*	1 (5.0%)		
*PTEN*	1 (5.0%)		
*SMAD4*	1 (5.0%)		
*TSC1*	1 (5.0%)		
*WT1*	1 (5.0%)		

SeC, sebaceous carcinoma.

**Table 3:** List of genes with frequent pathogenic mutations according to the molecular subtypes.

Gene	Luminal 1 (*n* = 7)	Luminal 2 (*n* = 3)	HER2 (*n* = 4)	All-Negative (*n* = 5)	Core Basal (*n* = 3)
*TP53*	5 (38.5%)	1 (7.7%)	3 (23.1%)	3 (23.1%)	1 (7.7%)
*SMARCA4*	3 (60%)	0 (0%)	1 (20%)	0 (0%)	1 (20%)
*FBXW7*	4 (100%) *	0 (0%)	0 (0%)	0 (0%)	0 (0%)
*NOTCH1*	0 (0%)	0 (0%)	1 (25%)	0 (0%)	3 (75%) †
*ATM*	1 (33.3%)	0 (0%)	1 (33.3%)	1 (33.3%)	0 (0%)
*BRAF*	2 (66.7%)	0 (0%)	1 (33.3%)	0 (0%)	0 (0%)
*PIK3CA*	2 (66.7%)	0 (0%)	1 (33.3%)	0 (0%)	0 (0%)
*BRCA1*	1 (50%)	0 (0%)	0 (0%)	1 (50%)	0 (0%)
*ERBB2*	2 (100%)	0 (0%)	0 (0%)	0 (0%)	0 (0%)
*HRAS*	1 (50%)	1 (50%)	0 (0%)	0 (0%)	0 (0%)
*MLH1*	1 (50%)	0 (0%)	0 (0%)	0 (0%)	1 (50%)
*MSH2*	0 (0%)	0 (0%)	1 (50%)	1 (50%)	0 (0%)
*MSH6*	0 (0%)	0 (0%)	1 (50%)	1 (50%)	0 (0%)
*NOTCH2*	1 (50%)	0 (0%)	1 (50%)	0 (0%)	0 (0%)
*VHL*	2 (100%)	0 (0%)	0 (0%)	0 (0%)	0 (0%)

* Significantly enriched in luminal A than in other subtypes. † Significantly enriched in core basal than in other subtypes.

**Table 4:** Mutations in suspected Muir–Torre syndrome patient.

Sample 29 SeC-O		Sample 12 SeC-EO	
*MLH1* mutation	VAF (%)	*MLH1* mutation	VAF (%)
p.M587Hfs * 6	45.5	p.M587Hfs * 6	60.16
Other pathogenic mutations		Other pathogenic mutations	
*FBXW7*	p.Q303 *	*NOTCH1*	p.W745 *
		*TP53*	p.R342P
		*TP53*	p.R175H

SeC-O, ocular sebaceous carcinoma; SeC-EO, extraocular sebaceous carcinoma; VAF, variant allele frequency.

## Data Availability

The main research data supporting the results of this study are included in Table 1 and Table 2 and Figure 1 and Figure 3. Other data can be made available upon reasonable request from the corresponding authors.

## Supplementary Materials

The following are available online at https://www.mdpi.com/article/10.3390/cancers13194810/s1, Figure S1: Kaplan–Meier analysis according to *RUNX1* and *ATM* mutation status, Figure S2: Gene ontology analysis in ocular sebaceous carcinoma, Figure S3: Gene ontology analysis in extraocular sebaceous carcinoma, Table S1: Information of targeted next-generation sequencing test (Axen Cancer Panel 2), Table S2: Determination of molecular subtypes of sebaceous carcinoma, Table S3: Gene ontology analysis of each ocular and extraocular sebaceous carcinoma, Table S4: Cases showing alterations in mismatch repair genes.
