# Supplementary material for: Targeted Sequencing Revealed Distinct Mutational Profiles of Ocular and Extraocular Sebaceous Carcinomas
Source: Cancers (Basel). 2021 Sep 26;13(19):4810. doi: 10.3390/cancers13194810 (PMC8508046; doi:10.3390/cancers13194810)
Supplement: Supplementary file 1 [file cancers-13-04810-s001.zip › cancers-1366838-supplementary.pdf]

cancers

MDPI

Supplementary materials

Targeted Sequencing Revealed Distinct Mutational Profiles of Ocular and Extraocular Sebaceous Carcinomas

Hee Young Na, Jeong Hwan Park, Sun Ah Shin, Sejoon Lee, Heonyi Lee, Heejoon Chae, HoKyung Choung, Namju Kim, Jin-Haeng Chung, Ji Eun Kim

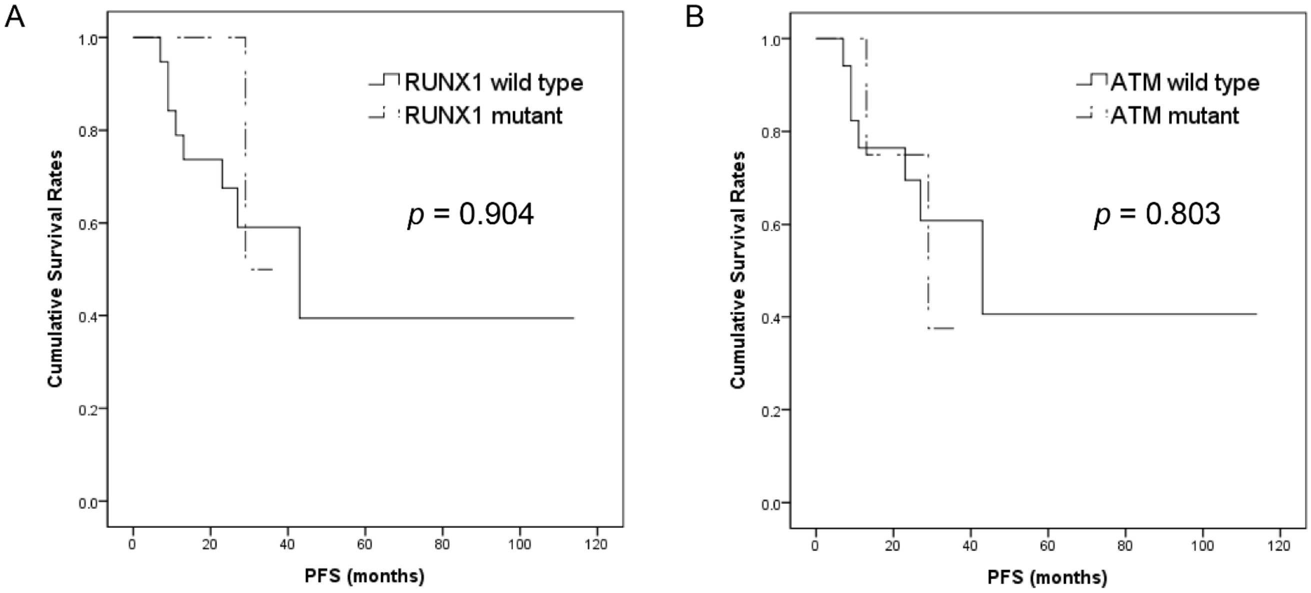

Figure S1. Kaplan–Meier analysis according to *RUNX1* and *ATM* mutation status. (**A**) *RUNX1* or (**B**) *ATM* mutation was not associated with shorter progression-free survival.

A

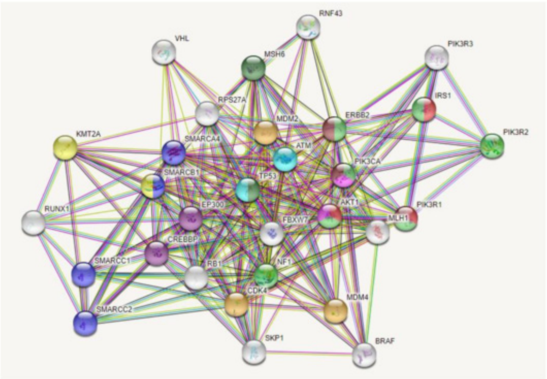

No.	ID	Description	Color
1	GO:0048009	insulin-like growth factor receptor signaling pathway	
2	GO:0006337	nucleosome disassembly	
3	GO:0014065	phosphatidylinositol 3-kinase signaling	
4	GO:2000615	regulation of histone H3-K9 acetylation	
5	GO:0043276	anoikis	
6	GO:0051095	regulation of helicase activity	
7	GO:0090399	replicative senescence	
8	GO:0071157	negative regulation of cell cycle arrest	
9	GO:0007221	positive regulation of transcription of Notch receptor target	
10	GO:0038128	ERBB2 signaling pathway	

B

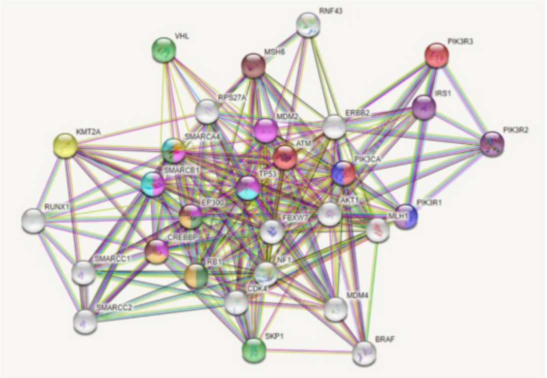

No.	ID	Description	Color
1	GO:0016303	1-phosphatidylinositol-3-kinase activity	
2	GO:0043560	insulin receptor substrate binding	
3	GO:1990756	ubiquitin ligase-substrate adaptor activity	
4	GO:0070577	lysine-acetylated histone binding	
5	GO:0002039	p53 binding	
6	GO:0050681	androgen receptor binding	
7	GO:0001046	core promoter sequence-specific DNA binding	
8	GO:0001102	RNA polymerase II activating transcription factor binding	
9	GO:0030971	receptor tyrosine kinase binding	
10	GO:0003684	damaged DNA binding	

Figure S2: Gene ontology analysis in ocular sebaceous carcinoma. (**A**) Gene ontology analysis based on biologic process. (**B**) Gene ontology analysis based on molecular function.

A

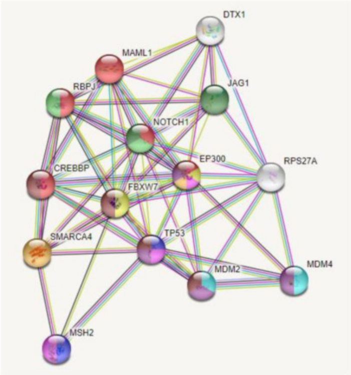

No.	ID	Description	Color
1	GO:0007221	positive regulation of transcription of Notch receptor target	
2	GO:0051095	regulation of helicase activity	
3	GO:0048505	regulation of timing of cell differentiation	
4	GO:1903025	regulation of RNA polymerase II regulatory region sequence-specific DNA binding	
5	GO:0042771	intrinsic apoptotic signaling pathway in response to DNA damage by p53 class mediator	
6	GO:2000737	negative regulation of stem cell differentiation	
7	GO:0071157	negative regulation of cell cycle arrest	
8	GO:0060765	regulation of androgen receptor signaling pathway	
9	GO:0006977	DNA damage response, signal transduction by p53 class mediator resulting in cell cycle arrest	
10	GO:0034644	cellular response to UV	

B

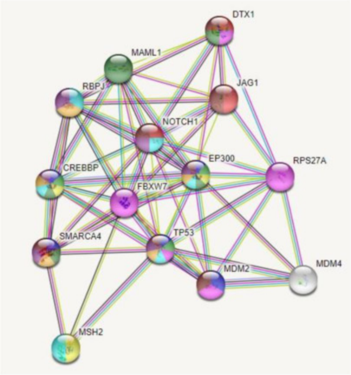

No.	ID	Description	Color
1	GO:0005112	Notch binding	
2	GO:0002039	p53 binding	
3	GO:0001085	RNA polymerase II transcription factor binding	
4	GO:0003684	damaged DNA binding	
5	GO:0031625	ubiquitin protein ligase binding	
6	GO:0003713	transcription coactivator activity	
7	GO:0003682	chromatin binding	
8	GO:0008134	transcription factor binding	
9	GO:0000976	transcription regulatory region sequence-specific DNA binding	
10	GO:0005102	signaling receptor binding	

Figure S3: Gene ontology analysis in extraocular sebaceous carcinoma. (**A**) Gene ontology analysis based on biologic process. (**B**) Gene ontology analysis based on molecular function.

Publisher's Note: MDPI stays neutral with regard to jurisdictional claims in published maps and institutional affiliations.

Copyright: © 2021 by the authors. Licensee MDPI, Basel, Switzerland. This article is an open access article distributed under the terms and conditions of the Creative Commons Attribution (CC BY) license (http://creativecommons.org/licenses/by/4.0/).
